# Role of mHealth applications for improving antenatal and postnatal care in low and middle income countries: a systematic review

**DOI:** 10.1186/s12913-017-2664-7

**Published:** 2017-11-07

**Authors:** Anam Feroz, Shagufta Perveen, Wafa Aftab

**Affiliations:** Department of Community Health Sciences, The Aga Khan University, Stadium Road, Karachi, 74800 Pakistan

**Keywords:** Mobile health, mHealth interventions, Antenatal care services, Postnatal care services, Low and middle income countries, Systematic review

## Abstract

**Background:**

From 1990 to 2015, the number of maternal deaths globally has dropped by 43%. Despite this, progress in attaining MDG 5 is not remarkable in LMICs. Only 52% of pregnant women in LMICs obtain WHO recommended minimum of four antenatal consultations and the coverage of postnatal care is relatively poor. In recent years, the increased cellphone penetration has brought the potential for mHealth to improve preventive maternal healthcare services. The objective of this review is to assess the effectiveness of mHealth solutions on a range of maternal health outcomes by categorizing the interventions according to the types of mHealth applications.

**Methods:**

Three international online electronic databases were searched between January 1, 2000 and January 25, 2016 to identify studies exploring the role of mHealth solutions in improving preventive maternal healthcare services. Of 1262 titles screened after duplication, 69 potentially relevant abstracts were obtained. Out of 69 abstracts, 42 abstracts were shortlisted. Full text of 42 articles was reviewed using data extraction sheet. A total of 14 full text studies were included in the final analysis.

**Results:**

The 14 final studies were categorized in to five mHealth applications defined in the conceptual framework. Based on our analysis, the most reported use of mHealth was for client education and behavior change communication, such as SMS and voice reminders [*n* = 9, 65%]. The categorization provided the understanding that much work have been done on client education and behavior change communication. Most of the studies showed that mHealth interventions have proven to be effective to improve antenatal care and postnatal care services, especially those that are aimed at changing behavior of pregnant women and women in postnatal period. However, little evidence exists on other type of mHealth applications.

**Conclusion:**

This review suggests that mHealth solutions targeted at pregnant women and women in postnatal period can improve preventive maternal healthcare services. However, there is a need to conduct more controlled-trials and quasi-experimental studies to strengthen the literature in this research area. The review recommends that mHealth researchers, sponsors, and publishers should prioritize the transparent reporting of interventions to allow effective interpretation of extracted data.

**Electronic supplementary material:**

The online version of this article (10.1186/s12913-017-2664-7) contains supplementary material, which is available to authorized users.

## Background

There has been an estimated 43% reduction in the number of maternal deaths globally, from 1990 to 2015 [1, 2]. The sluggish progress is evident in the current “countdown to 2015” report, which stated that only nine out of 74 countries with the highest Maternal Mortality Rates (MMRs) were on target to meet Millennium Development Goal five (MDG 5) [3]. The reasons for the slow progress in meeting MDG 5 in most countries is related to limited access to preventive maternal health services, poor administration, poor logistical and technical ability, insufficient financial assets and dearth of skilled health personnel [4]. While moving from MDG 5 to Sustainable Development Goals 3 (SDGs), reducing preventable maternal morbidity and mortality still remains a prioritized health agenda.

In low and middle income countries (LMIC), women face a lifetime risk of maternal death of one in 160, as compared with 1 in 3700 for women living in high income countries (HIC) [5]. Most women die because of complications during and following pregnancy including infections, pre-eclampsia, eclampsia and post-partum hemorrhage. Most of these complications are preventable and account for nearly 75% of all maternal deaths [6].

Antenatal care (ANC) and postnatal care (PNC) interventions have proven to be key health interventions to decrease maternal mortality. Studies in Tanzania and Ethiopia have proven the ability of ANC and PNC to reduce maternal mortality [7–9]. Improving maternal health outcomes requires reinforcement of prevailing evidence-based practices that include World Health Organization (WHO) recommended number of ANC (minimum of four ANC visits) and PNC visits. The timing and optimal number of PNC visits, particularly in resource-limited setting, is an area of debate. However, it is suggested that women should have at least one or more postnatal visits within 2 days of delivery [7, 8]. In LMICs, only 52% of pregnant women obtain the WHO suggested minimum of four antenatal visits [6]. The coverage of PNC in LMIC is relatively poor and, therefore, nearly 40% of women develop complications following delivery and an almost 15% encounter potentially life-threatening complications [7, 10]. The poor ANC and PNC attendance account for substantial number of these preventable deaths [11].

Surprisingly, many countries with limited print or internet resources have gained substantial level of cell phone penetration. According to ‘International Telecommunication Union 2015’ figures, cell phone subscriptions have reached over 7 billion worldwide and in LMICs mobile penetration has reached over 90%. [12, 13]. The increased penetration of cellphone over recent years has brought the potential for mobile health to improve ANC and PNC services by addressing issues such as low literacy level, large geographical distances to services, social marginalization, unskilled human resource, and poor financial resources [12, 13]. The increasing proliferation of mobile technology is bringing up new opportunities to permit safe, accessible, coordinated and effective maternal health care [6]. There are numerous models of mHealth interventions being used to support pregnant mothers through safe pregnancy and childbirth in LMICs [6, 13]. Mobile health or mHealth, refers to the use of mobile phones, personal digital assistants (PDAs), patient monitoring devices and other Information and Communication Technologies (ICT) to support and deliver health and healthcare services.

Many initiatives have been taken to plot the strength of the evidence regarding mHealth for improving preventive maternal healthcare services including ‘Phil Brick’s gap analysis for mHealth Alliance’, stakeholder interviews, pooled review by Noordam et al., and a systematic review by Tamrat and Kachowski [14–16]. However, none of the reviews explored the effectiveness of mHealth solutions on a range of maternal health outcomes by categorizing the interventions according to the types of mHealth applications. Given the potential significance of the context, a review on exploring the role of mHealth applications for improving antenatal and postnatal care in low and middle income countries may provide valuable insights in to pertinent issues.

Labrique and colleagues identified 12 mHealth applications to respond to various health issues [17]. Few projects involve one application and other projects may include two or more mHealth applications for addressing specific health issue. The classification of 12 mHealth applications as per Labrique and colleagues is illustrated in Table 1. The same framework is used to guide the conduct of this systematic review.

**Table 1:** Twelve common mHealth applications

1. Client education and behavior change communication (BCC)
2. Sensors and point-of-care diagnostics
3. Registries/ vital events tracking
4. Data collection and reporting
5. Electronic health records
6. Electronic decision support (information, protocols, algorithms, checklists)
7. Provider to provider communication (user groups and consultation)
8. Provider work planning and scheduling
9. Provider training and education
10. Human Resource management
11. Supply chain management
12. Financial transactions and incentives

## Objective

The objective of this review is to assess the effectiveness of mHealth solutions on a range of maternal health outcomes by categorizing the interventions according to the types of mHealth applications.

## Methods

An electronic systematic literature search was carried out on role of mHealth solutions in improving preventive maternal healthcare services in LMIC. The protocol was registered in the ‘International Prospective Register for Systematic Reviews’ (PROSPERO) CRD42016035503 [18]. The studies involving target groups of women who were in antenatal and postnatal period and healthcare workers by which mHealth interventions delivered to these groups were included. LMICs were shortlisted according to the World Bank’s 2016 Country Classification [19]. Issues regarding use of mobile phones are shared across many LMIC, therefore these studies are more comparable than those conducted in HIC. The inclusion and exclusion criterion applied to this review is illustrated in Table 2.

**Table 2:** Eligibility Criteria

Attribute	Inclusion Criteria	Exclusion Criteria
Population	Studies involving target groups of women in antenatal and postnatal period and healthcare workers through which interventions were delivered to these groups.	Studies involving target groups women, adolescent females and girls over the age of 5 years who are not pregnant and have not recently given birth, newborn and children and decision makers and facility managers that are not directly involved in patient care
Intervention	Studies involving mHealth interventions	Studies involving other ICT interventions, ART compliance reminders, EmONC coverage, managerial and financial level interventions, physical mobile clinics and teleconsultations
Outcome	Outcomes demonstrating improvement in preventive maternal healthcare services	Outcomes demonstrating skilled birth attendants, emergency care, quality of life, immunization coverage, cost-effectiveness of intervention, child development and others
Setting	Studies conducted or implemented in LMICs	Studies conducted or implemented in high income countries
Type of studies	Original studies, case studies, cross-sectional studies, case control studies, randomized controlled trials, quasi experimental studies, before and after (pre-post studies), qualitative formative studies, systematic reviews and clinical control trials	Commentaries, editorials, symposium proceedings and irretrievable documents
Language	Studies available in English Language	Studies which were not available in English translation
Time period	Studies published between 1 January, 2000 to 25 January, 2016	Studies published before 1 January, 2000 and after 25 January, 2016

### Information sources and search strategy

Three international electronic databases PubMed, CINAHL Plus and Cochrane were searched using detailed search strategy. Cross referencing of systematic reviews was also undertaken to identify relevant articles using hand search. The database searches were performed by two researchers independently. The search terms were grouped in to four major categories of interest: population, intervention, outcome and settings. In order to make uniform search terms, the Medical Subject Headings (MeSH) were utilized wherever applicable. The search strategy was also piloted. The search strategy applied to this review is illustrated in Table 3.

**Table 3:** Search Strategy

Population	“Pregnancy”[Mesh] OR pregnan* OR pregnant women OR pregnant mother* OR gestational mother* OR women OR maternal age women OR matern* OR “Healthcare providers” [Mesh] AND
Intervention	mHealth OR m–Health OR mobile health OR mobile telehealth care OR mobile telemedicine OR mcare OR mobile phone OR mobile devic* OR mobile technology OR mobile commmunication OR satellite phone OR communication satellite OR enterprise digital assistants OR cell phone OR cellular phone OR personal digital assistant* OR PDA OR mobile tablet computers OR smart-phone OR smartphone OR podcast Or pod-cast OR apps OR mobile applications OR text messag* OR short messag* OR short message service OR SMS OR multimedia messag* OR MMS OR texting OR messag* OR text* OR multimedia technol* OR multi-media messag AND
Outcome	Reproductive health services OR maternal welfare OR maternal healthcare OR maternal health OR maternal service* OR reproductive health OR reproductive service* OR pre-natal care OR pre-natal visit* OR prenatal care OR prenatal visit* OR antenatal care OR antenatal visit* OR Postnatal Care OR postpartum program* OR safe motherhood OR perinatal complications OR postnatal complication OR perinatal care OR essential preventive maternal health services AND
Setting	Developing country OR developing nation OR least developed country OR least developed nation OR less developed nation OR third world country OR third world nation OR under developed country OR remote region OR low and middle income country OR under developed nation OR low and middle income nation OR Armenia OR Moldova OR Bangladesh OR Morocco OR Bhutan OR Myanmar OR Bolivia OR Nicaragua OR Cabo Verde OR Nigeria OR Cameroon OR Pakistan OR Congo, Rep OR Papua New Guinea OR Cote d’Ivoire OR Philippines OR Djibouti OR Samoa OR Egypt, Arab Rep. OR Sao Tome and Principe OR El Salvador OR Senegal OR Georgia OR Solomon Islands OR Ghana OR Sri Lanka OR Guatemala OR Sudan OR Guyana OR Swaziland OR Honduras OR Syrian Arab Republic OR India OR Tajikistan OR Indonesia OR Timor-Leste OR Kenya OR Ukraine OR Kiribati OR Uzbekistan OR Kosovo OR Vanuatu OR Kyrgyz Republic OR Vietnam OR Lao PDR OR West Bank and Gaza OR Lesotho OR Yemen, Rep. OR Mauritania OR Zambia OR Micronesia, Fed. Sts.

### Study selection

**Fig. 1:**
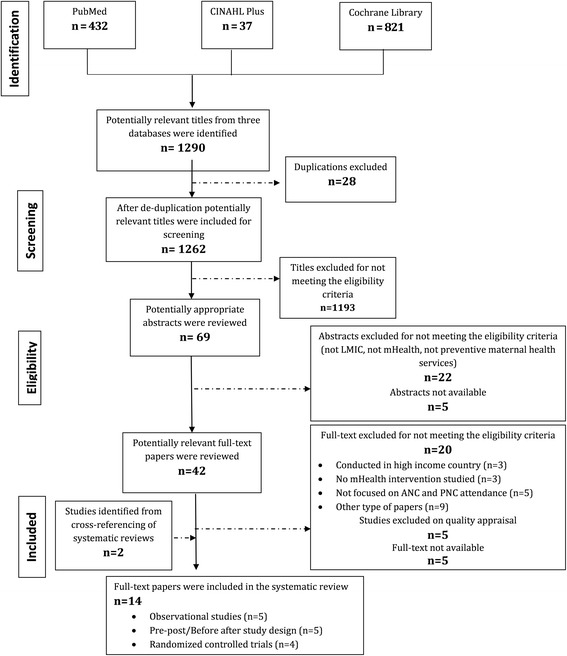
PRISMA Flow Diagram for Database Search of Studies

The resulting studies were first screened by titles, then by abstract, and finally by full text to progressively eliminate studies not meeting the inclusion criteria. Database searches identified a total of 1290 studies initially. After de-duplication, 1262 records were screened by titles. After title screening, 69 records were screened by abstracts. Full texts of remaining 42 studies were reviewed to determine if they fulfill the inclusion criteria. All the potentially relevant full-texts were assessed for quality using Cochrane Risk of Bias Assessment Tool and Newcastle-Ottawa-Quality Assessment Scale [20, 21]. Two additional relevant studies were also identified from cross-referencing of systematic reviews. Finally, 14 studies were selected and used for the purpose of this review [22–35]. The results of study screening and selection are illustrated in Fig. 1.

### Data collection process

Two reviewers screened the titles, abstracts and full text of selected studies against the inclusion and exclusion criteria independently using a customized data extraction form. Any discrepancies between the two reviewers in this process were discussed with the third reviewer until consensus was reached. The sheet was designed on MS Excel spreadsheet using existing literature and subject experts’ opinion. The information extracted included author, year of publication, study title, purpose of study, type of paper/study design, health issue studied, study method, interventional group, control group, primary or secondary intervention for improving preventive maternal healthcare services, intervention frequency, key study outcomes – improved ANC and PNC services, and quality appraisal of included studies. Later, Labrique and colleagues framework was utilized to organize the data. The summary of included studies on mHealth interventions to improve preventive maternal healthcare services is provided as an appendix [see Additional file 1].

### Quality assessment of included studies

Risk of bias was thoroughly examined for all the included randomized controlled trails (RCTs) (*n* = 4) using the Cochrane Risk of Bias Assessment Tool [20]. This tool was used to evaluate risk of bias in each study by evaluating a study’s allocation sequence generation (randomization), allocation concealment, blinding, incomplete data, selective reporting and other potential threats to the study’s validity. According to the scale, a low risk of bias is the best possible rank indicating higher quality. All the RCTs (*n* = 4) generally performed well and attained low risk of bias on Cochrane Risk of Bias Assessment Tool.

The quality of non-randomized studies was examined using the ‘Newcastle-Ottawa-Quality Assessment Scale’ [21]. This scale was used to assess the quality of observational (*n* = 5) and pre-post study design (n = 5) by assessing potential sources of bias in the selection and comparability of participants, the assessment of outcomes and the duration and adequacy of follow-up. For non-randomized studies, the scores were awarded out of nine possible points, with higher score indicating higher quality. All the non-randomized studies generally performed well and attained an average score of eight out of nine possible points. Five non-randomized studies attained a score of eight out of nine. Four studies attained a score of seven out of nine. Remaining one study scored five out of nine possible points. The quality assessment of all included studies is provided in the data extract sheet [see Additional file 1].

## Results

**Fig. 2:**
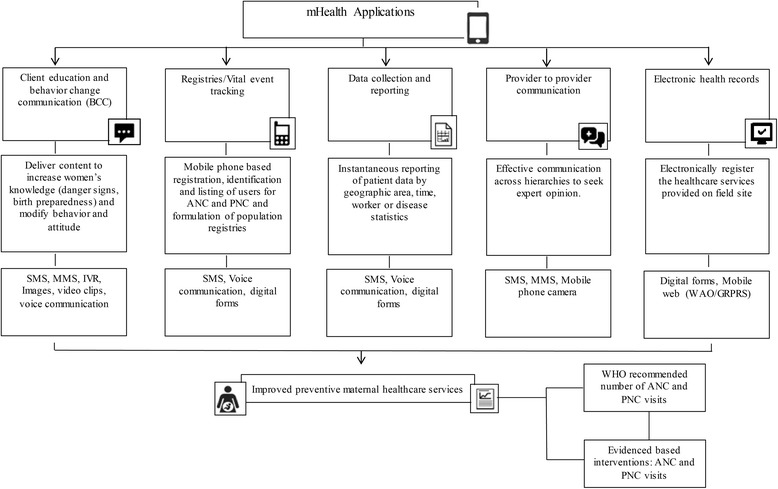
Conceptual Framework on mHealth Applications for Preventive Maternal Healthcare Services

The review is stated according to the Preferred Reporting Items for Systematic Reviews and Meta-analyses (PRISMA) guidelines. The data from 14 final studies only fit in to five main mHealth applications defined in the Labrique and colleagues framework which include ‘client education and behavior change communication, registries/vital event tracking, data collection and reporting, provider to provider communication, and electronic health records’. All of these applications have been operationalized using various mobile phone functions that include “short message service (SMS), multimedia messaging service (MMS), Interactive voice response (IVR), voice communication, video clips, images, audio clips and packages, apps, mobile phone camera, digital forms and mobile web” [17]. The conceptual framework is adapted to elaborate the potential of mHealth applications for preventive maternal healthcare services (ANC and PNC). The adapted framework is illustrated in Fig. 2.

### Types of studies

Out of fourteen studies which were included, four were randomized controlled trials, five were observational studies (qualitative formative study, cross sectional study) and remaining five were pre-post design studies. All studies included in the review were published within the time period of 2000 to 2016.

### Types of mHealth interventions

**Fig. 3:**
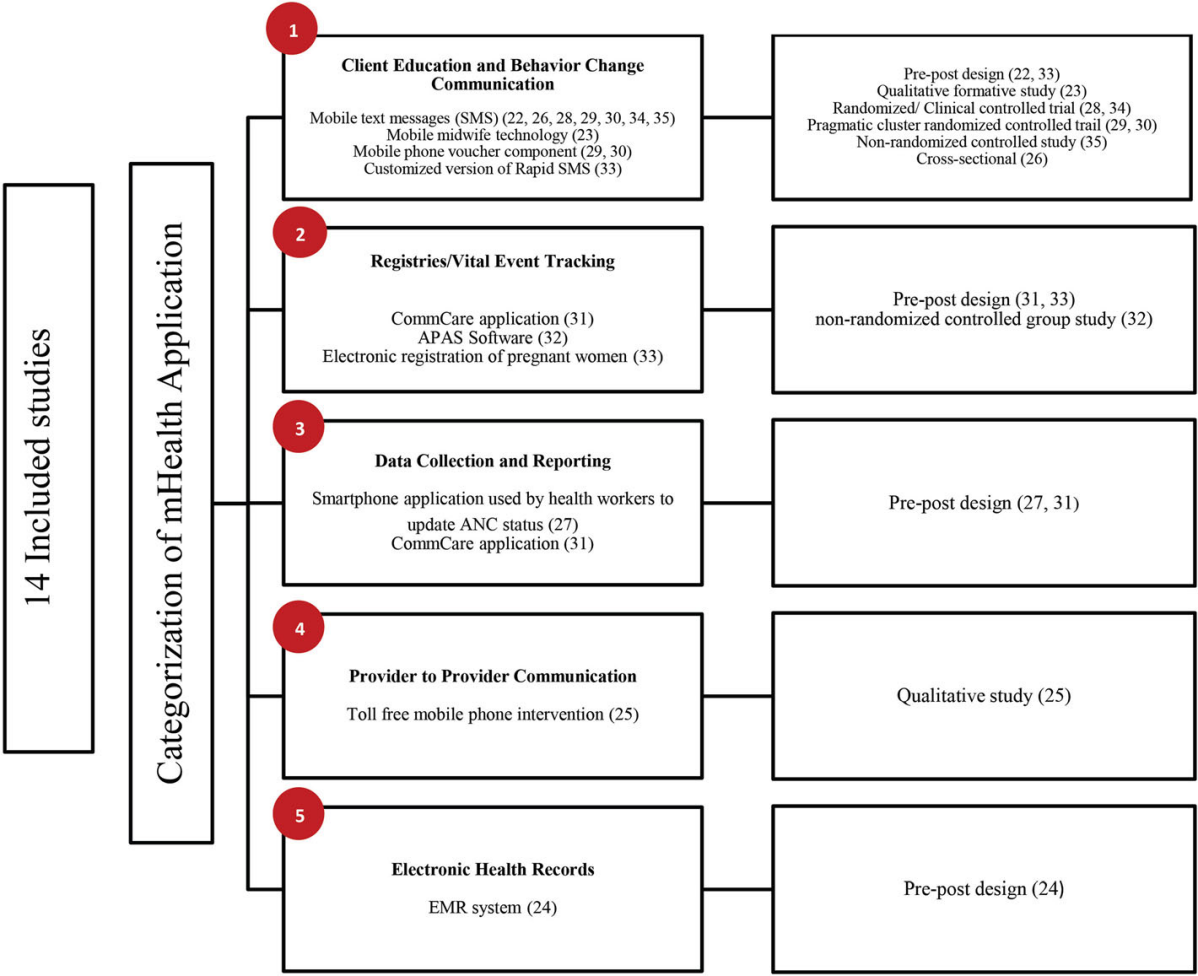
Classification of the Included Studies Based on the Types of mHealth Interventions Used

The final studies were classified according to the types of mHealth applications. While few studies addressed one mHealth application, many addressed multiple applications. Most of the studies were assigned in more than one category if the intervention was multi-faceted. When the studies were broken down into types of mHealth applications, the total adds up to 16 types of mHealth interventions. The final studies were broadly categorized in five main applications which include client education and behavior change communication (*n* = 9) [22, 23, 26, 28–30, 33–35], registries/vital event tracking (*n* = 3) [31–33], data collection and reporting (*n* = 2) [27, 31], provider to provider communication (*n* = 1) [25], and electronic health records (n = 1) [24]. The results of the classification exercise are illustrated in Fig. 3.

#### Client education and behavior change communication

In Njoro Division, a randomized controlled trial evaluated the impact of mobile telephone support on antenatal attendance. A group of 191 pregnant women were regularly given advice and prompts regarding pregnancy care and scheduled antenatal visits through mobile phone; whereas, the other groups of 206 pregnant women were provided usual care to continue antenatal visit. Positive association was found among women in intervention group and the number of ANC visits (96.4% in intervention group and 92.3% in the control group, *P* value: 0.002) (Fig. 3). [34]

In Zanzibar, a pragmatic cluster randomized controlled trial was conducted in primary health facilities. Twenty-four healthcare facilities were randomized to either intervention group or usual care group. The intervention consists of two components which include SMS and mobile phone voucher. The SMS component was used to send appointment reminders and educational messages to women regarding ANC and PNC. For the SMS component, web-based system was developed to register women with their phone numbers. The mobile phone voucher component allowed pregnant women (mothers) to directly communicate with primary healthcare providers. This allowed access to emergency obstetric care through improved communication (Fig. 3). [29]

A Similar pragmatic cluster randomized controlled trial was conducted in primary healthcare facilities of Zanzibar. The primary outcome measure of the trial was four or more ANC visits. The intervention consisted of two components SMS and mobile phone voucher component. The intervention was related with an improvement in ANC visits in the intervention group. In the intervention group, 44% of women attained four or more ANC visits versus 31% in the comparison group (OR, 2.39; 95% CI 1.03–5.55) (Fig. 3) [30].

In Ghana, a qualitative formative study was conducted to explore the role of Mobile Midwife technology in the lives of pregnant mothers. The Mobile Midwife technology routinely sent reminders to pregnant mothers of planned ANC visits and their related significance. Nineteen individual interviews and three Focus Group Discussions (FGDs) were conducted and it was found that women gradually gained trust in the Mobile Midwife technology. Women verbalized that the frequency of ANC visits increased after the introduction of mobile technology (Fig. 3) [23].

In a rural area of Tamil Nadu, India, a pre-post study was conducted to evaluate whether mobile text messaging service is a feasible mode of raising knowledge level regarding Maternal and Child Health (MCH) services. Data was obtained using a questionnaire in three phases; a) baseline assessment, b) intervention: MCH related messages were sent, c) end line assessment. It was found out that 45 (37.5%) individuals knew about minimum number of antenatal visits during pregnancy after receiving text messages, as compared to 12 (10%) individuals before receiving text messages (*P* value <0.05, 95% CI: 0.16–0.38) (Fig. 3) [22].

In Rawanda, a pre-post study was conducted to monitor pregnancy and reduce maternal and child deaths. An innovative SMS- based alert system (RapidSMS-MCH) was developed to permit interactive communication between CHW and mother-infant pairs. A total of 432 CHWs were trained and equipped with mobile phones. A total of 35,734 SMS were sent by CHWs. A total of 11,502 pregnancies were monitored and 362 SMS life threatening events were registered. It was found out that CHWs were more pro-active in finding new pregnant women and following them up as a result of reminders sent to the mothers (Fig. 3) [33].

In South Africa, a mixed-method study was conducted to increase antenatal awareness and health knowledge by disseminating text messages. First, a controlled trial was conducted in primary health facility of Cape Town. The intervention group (*n* = 102) received SMS and the control group (*n* = 104) received no text messages. The text messages included antenatal health information in their preferred language. A baseline knowledge questionnaire was administered prior to the intervention and after the intervention with additional health related behavior questions. No statistically significant difference was attained between the two groups (*P* > 0.05). A focused group of seven participants from the intervention group was also performed and it was found out that SMS acted as a reminder and a source of motivation for the pregnant mothers (Fig. 3) [28].

In Nigeria, a study was conducted to compare the postnatal Clinic attendance among the intervention group (*n* = 1126) and the historic control group (*n* = 971). Text message reminders were delivered to postnatal mothers in the intervention group. It was found out that the intervention group, receiving text message appointment reminders, were 50% less likely to fail to attend (FTA) their postnatal appointment (RR of FTA 0.50; 95% CI, 0.32–0.77; *P* = 0.002) (Fig. 3) [35].

In Nigeria, a population based survey was conducted to investigate if women with restricted mobile phone access have differential odds of maternal knowledge and service utilization as compared to female mobile phone. Multivariate logistic regressions were used to calculate the odds of maternal knowledge and service utilization by mobile phone usage. Findings showed that women without mobile phone access had significantly lower odds of ANC utilization (OR = 0.48, 95%CI: 0.36–0.64) compared to female mobile phone users. Also, women without mobile phone access had significantly lower knowledge of ANC attendance (OR = 0.46, 95%CI: 0.36–0.59) compared to female mobile phone users. No differences were observed by mobile phone users in uptake of postnatal services (Fig. 3) [26].

#### Registries/vital event tracking

In Rawanda, a pre-post study was conducted to monitor pregnancy and reduce maternal and child deaths. An innovative SMS-based alert system (RapidSMS-MCH) was developed to ensure Electronic registration of pregnant women through text messages by community health workers (CHWs). 81% of the annual pregnancies in the district were registered in the system. Reporting compliance among CHWs was 100% (Fig. 3) [33].

In northern Nigeria, a pre-post study was conducted to assess whether the initiation of the CommCare mobile phone application had an effect on the quality of ANC services provided by lower-level cadre. The CommCare application guides CHWs to register new clients by collecting demographic information, past medical history, height, weight and other medical information. Also, the application helps in following-up clients on subsequent visits. Through the introduction of CommCare, the quality score improved from 13.3 at baseline to 17.2 at end line (*P* < 0.0001) (Fig. 3). [31]

In western Kenya, an evaluation study was conducted to assess the impact of mobile health system on antenatal and postnatal attendance. CHWs (*n* = 20) were interviewed to assess the adherence to ANC and PNC following registration of 800 women in to mobile health system (APAS). All CHWs communicated that APAS help them track vital events efficiently, as compared to paper based tracking system (Fig. 3) [32].

#### Data collection and reporting

In northern Nigeria, a before-after study was conducted to assess whether the initiation of the CommCare mobile phone application had an effect on the quality of ANC services provided by lower-level healthcare workers. The CommCare application guides CHWs to collect client data (medical information) in real time. The application helps CHWs to collect information during the examination (fetal heart rate, maternal and fetal danger signs - if any) and lab results (protein or glucose in urine, Hb levels, malaria test, UTI test). Through the introduction of CommCare, the quality score improved from 13.3 at baseline to 17.2 at end line (*P* < 0.0001) (Fig. 3) [31].

In Thai-Myanmar border area, a study was conducted to evaluate the application of mobile phone integrating in to health system to improve ANC for the undeserved population. ‘A module containing web-based and mobile technology system was developed to generate ANC visit schedule dates in which CHW could cross-check, identify and update the mother’s ANC status at the healthcare facility or at the household location when performing home visit’. Findings showed that the module enhanced ANC coverage in the Thai Myanmar border area by developing better procedures of data collection and reporting (Fig. 3) [27].

#### Provider to provider communication

In rural Bangladesh, a qualitative research was conducted to assess the impact of toll free mobile communication in improving communication for maternal and neonatal complication. Focused group discussions, in-depth interviews and semi-structures interviews were conducted with community skill birth attendants and pregnant women. Women verbalized that once a complication is reported over phone, SBAs either visit to mothers by themselves or advise them for direct referral. More than 80% SBAs communicated with solution linked group to receive prompt help. The solution linked group involved eight experts from the field of maternal and child health. Women verbalized that SBAs have become competent in managing complications due to effective communication with solution linked group (Fig. 3) [25].

#### Electronic health records

In rural Kenya, a study implemented a ‘novel cloud-based electronic medical record system’ in a MNCH outpatient setting and assessed its impact on improving completeness of data collected by healthcare staff. Findings showed significant improvements in completeness of the antenatal record were reported through implementation of Electronic Medical Records (EMR) -based data verification (Fig. 3) [24].

### Type of outcomes examined

#### Antenatal care services

Four studies examining ANC services were RCTs [28–30, 34]. All studies used text message (SMS) appointment reminders and antenatal education for pregnant women and two also provided the women with mobile phone voucher component to contact their health worker, if needed [29, 30]. All studies found a positive association between women in intervention group receiving mHealth intervention and the number of ANC visits. Five studies examined ANC services before and after implementation of mHealth applications for improved patient electronic records, data collection and reporting, vital event tracking and automated appointment reminders; these studies similarly found a statistically significant improvement in on-time ANC attendance, data completeness, vital event tracking and registries following implementation [22, 24, 27, 31, 33]. Two qualitative studies conducted in LMICs evaluated the impact mobile midwife technology and toll free mobile phone communication on quality of ANC and health advice; women in both studies verbalized that ANC visits have improved after the introduction of mobile technology and SBAs have become competent in managing complications due to communication with ‘solution linked group’ [23, 25]. The remaining two studies examining ANC services found some self-reported behavior change from both patients and health workers after using APAS software and RapidSMS application [26, 32].

#### Postnatal care services

Only few studies evaluated the impact of mHealth interventions on postnatal care services [26, 32, 35]. One study examined the effect of mHealth intervention on PNC attendance by comparing an intervention group with a historical control group and found that the intervention group, receiving text message appointment reminders, were 50% less likely to fail to attend their visit (*P* = 0.002) [35].

## Discussion

All included studies showed some evidence that mHealth interventions can play a major role in improving a range of maternal health outcomes. Most of the studies took place in East Asia and Sub–Saharan Africa, while some were undertaken in Middle East and South Asia. mHealth solutions are increasingly being utilized to increase the quality of pre- and post-pregnancy care, and as a way of collecting pregnancy data. A few studies reported that mHealth interventions, particularly those delivered through SMS, were associated with improved utilization of preventive maternal healthcare services, including uptake of recommended ANC and PNC services. In most studies, authors did not describe the basis of their intervention, in terms of its pathways through which it would be delivered to target groups. Moreover, the studies did not utilize a common taxonomy for explaining the type and purpose of the mHealth intervention. In addition, several studies combined multiple mHealth interventions [31, 33], make it difficult to determine to what extent each intervention brought the expected outcome.

To aid interpretation, Labrique and colleagues framework was adapted for categorizing the mHealth interventions according to their purpose, as previously described (Fig. 1). Based on our analysis, the most reported use of mHealth was for client education and behavior change communication*,* such as SMS reminders, [22, 23, 26 28, 29, 30, 33 34, 35]. This was followed by registries and vital event tracking, to enable ANC and PNC registration or reporting of health indicators [31–33]. The other observed categories were mHealth as a Data collection and reporting, mainly to ensure data recording and completeness [27, 31]; as a provider to provider communication platform, to access support from care providers [25]; and as an electronic health record system to ensure availability of health information systems [24]. The categorization of the studies in to various mHealth applications provided the understanding that the strongest evidence exists on client education and behavior change communication mHealth application. However, little evidence exists on other type of mHealth applications such as, registries and vital events tracking, data collection and reporting, provider to provider communication and electronic health records. Thus, there is an extensive need to perform research in overlooked areas to strengthen the evidence base.

This review with comprehensive search strategy, analyses both ANC and PNC attendance indicators as two most important components of preventive maternal healthcare services. However, much of the literature reported impact of mHealth solutions on antenatal attendance and only a few assessed the effect of mHealth interventions on postnatal attendance. ANC and PNC, both have long been regarded as critical for detecting a number of women who are at more risk of poor pregnancy outcomes [8]. Especially, a strong positive association between improved PNC services and reduction in maternal mortality is established. As improved PNC services prevent women from life- threatening fatal pathologies (post-partum hemorrhage, post-partum depression, etc.). It is advocated that PNC should be given importance by using mHealth solutions to identify post-pregnancy risks in women who may be predisposed to a range of potentially fatal pathologies.

As with most systematic reviews in the field of mHealth, this review is restricted by the difficulty of analyzing complex intervention studies and the variable description of interventions across studies. More studies are needed to refine the current work with a larger body of evidence and to establish how best to integrate it with the published existing frameworks. The heterogeneity of the interventions and study outcomes restricted the interpretation through meta–analyses. The current literature contains many studies describing the use of mHealth for improving preventive maternal healthcare services in LMICs but only some have vigorously assessed the impact of mHealth solutions. Overall, most studies included in this review was of moderate quality, highlighting the significance of increasing the methodological rigor of future research. For randomized trials, there is need for allocation concealment and adequate blinding of outcomes, while the quality of observational studies can be improved via prospective research designs and adjustment for confounding variables.

As a result of methodological limitations and the small number of studies meeting the inclusion criteria, further randomized controlled trials and scaling up of pilot studies are needed to explore the potential of mHealth for improving preventive maternal healthcare services.

## Conclusion

The review concludes that mHealth solutions targeted at pregnant women and women in postnatal period can improve antenatal and postnatal care services in LMICs. There is a growing body of evidence highlighting the effectiveness of mHealth interventions on a range of maternal health outcomes in LMIC, but overall the available literature is weak and the results, in most cases, are too inconsistent to enable robust conclusions to be drawn about impacts. However some supportive literature exists with respect to the use of SMS for increasing ANC and PNC services. In particular, RCTs with economic, clinical and long-term patient-centered outcomes are suggested. This review recommends that mHealth researchers, sponsors, and publishers should prioritize the transparent reporting of interventions in terms of their aims, contexts, pathway of delivery and assumed mechanisms of impact to allow effective interpretation of extracted data. As low cost smartphones start to penetrate in these regions, a new generation of mobile applications are now emerging, which will also require evidence-based methods to establish their safety, efficacy and societal impacts.

## Additional file

Additional file 1: Summary of study findings. Description of included studies and quality appraisal (DOCX 21 kb)

## Abbreviations

ANC: Antenatal care
ART: Antiretroviral Therapy
CHWs: Community Health Workers
EmONC: Emergency Obstetric and Neonatal Care
EMR: Electronic Medical Records
FGDs: Focus Group Discussions
HIC: High Income Countries
ICT: Information and Communication Technologies
IVR: Interactive Voice Response
LMIC: Low and Middle Income Countries
MCH: Maternal and Child Health
MDG: Millennium Development Goal
MeSH: Medical Subject Headings
MMR: Maternal Mortality Rate
MMS: Multimedia Messaging Service
PDA: Personnel Digital Assistants
PNC: Postnatal Care
PRISMA: Preferred Reporting Items for Systematic Reviews and Meta-analyses
RCTs: Randomized Control Trials
SBA: Skilled Birth Attendants
SDGs: Sustainable Development Goals
SMI: Safe Motherhood Initiative
SMS: Short Messaging Service
WHO: World Health Organization
