# Supplementary material for: Role of mHealth applications for improving antenatal and postnatal care in low and middle income countries: a systematic review
Source: BMC Health Serv Res. 2017 Nov 7;17:704. doi: 10.1186/s12913-017-2664-7 (PMC5678803; doi:10.1186/s12913-017-2664-7)
Supplement: Additional file 1: — Summary of study findings. Description of included studies and quality appraisal (DOCX 21 kb) [file 12913_2017_2664_MOESM1_ESM.docx]

Summary of study findings

| First author, year | Title of publication | Purpose of study: | Type of paper/ Study design: | Health issue studied | Interventional group | Control  group | Primary or secondary intervention | Intervention frequency | Key study outcomes | Quality appraisal |
| --- | --- | --- | --- | --- | --- | --- | --- | --- | --- | --- |
| Datta, 2014 [22] | A study to assess the feasibility of Text Messaging Service in delivering maternal and child healthcare messages in a rural area of Tamil Nadu, India | To evaluate whether mobile Text Messaging Service is a feasible mode of raising knowledge regarding MCH and to explore issues related to mobile text messages as a mode of health education | Pre-post study design | Maternal and Child Health (MCH) | - | - | Mobile text messages with standard MCH practices were sent to each respondent. A total of 10 standard messages were sent to all 120 respondents in both Tamil and English font. | One message per day, these messages were brief to fit the standard text message limit of a regular mobile handset | It was found out that 45 (37.5 %) individuals knew about minimum number of antenatal visits during pregnancy after receiving text messages, as compared to 12 (10%) individuals before receiving text messages (P value < 0.05, 95 percent CI: 0.16-0.38) | 7/9 |
| Entsieh, 2015 [23] | Learning the ABCs of pregnancy and newborn care through mobile technology | To investigate the role that Mobile Midwife technology has played in the lives of pregnant and nursing mothers in Awutu Senya District, Ghana | Qualitative formative study | Maternal Health | - | - | Mobile midwife technology |  | It was found out that women gradually gained trust in the Mobile Midwife technology. Women verbalized that the frequency on ANC visits increased after the introduction of mobile technology | 8/9 |
| Haskew, 2015 [24] | Implementation of a cloud-based electronic medical record for maternal and child health in rural Kenya | To describe the implementation of a novel cloud-based EMR system for maternal and child health in Western Kenya. | Pre-post study | Maternal and Child Health (MCH) | - | - | EMR system | not mentioned | Findings showed significant improvements in completeness of the antenatal record were reported through implementation of ‘EMR-based data verification’ | 8/9 |
| Huq, 2014 [25] | Toll free mobile communication: overcoming barriers in maternal and neonatal emergencies in Rural Bangladesh | To describe the attributes of the participants that supported to initiate the mobile phone pathway. | Qualitative study | Maternal and Child Health (MCH) | - | - | Toll-free mobile phone intervention | not mentioned | More than 80% SBAs communicated with solution linked group to receive prompt help. Women verbalized that SBAs have become competent in managing complications due to effective communication with solution linked group | 8/9 |
| Jennings, 2015 [26] | Disparities in mobile phone access and maternal health service utilization in Nigeria: a population-based survey | To examine if women with limited mobile phone access have differential odds of maternal knowledge and health service utilization as compared to female mobile phone users who are currently eligible to participate in maternal mHealth programs. | Survey (cross-sectional) | Maternal and Child Health (MCH) | - | - | - | - | Findings showed that women without mobile phone access had significantly lower odds of antenatal care utilization (OR=0.48, 95%CI: 0.36-0.64) compared to female mobile phone users. Also, women without mobile phone access had significantly lower knowledge of antenatal care attendance (OR=0.46, 95%CI: 0.36-0.59) compared to female mobile phone users. No differences were observed by mobile phone users in uptake of postnatal services | 7/9 |
| Kaewkungwal, 2010 [27] | Application of smart phone in "Better Border Healthcare Program": a module for mother and child care | To assess the application of cell phone integrating into the healthcare system to improve antenatal care (ANC) and expanded programme on immunization (EPI) services for the under-served population in border area. | Before/after study design | Antenatal care attendance and childhood immunization | - | - | Smartphone application used by health workers to update antenatal and immunization status when outside clinic and SMS reminders for both health workers and mothers. Tools used: custom Mother and Child Care Module (MCCM) | Appointment reminders a few days prior to scheduled appointment | Findings showed that the module enhanced ANC coverage in the Thai Myanmar border area by developing better procedures of data collection and reporting | 8/9 |
| Lau, 2014 [28] | Antenatal health promotion via short message service at a Midwife Obstetrics Unit in South Africa: a mixed methods study | To increase antenatal health knowledge and awareness by disseminating text messages about clinic procedures at antenatal visits, and how to be healthy during pregnancy. | Controlled clinical trial | Antenatal care attendance | Intervention group (text messages) n=102 | Control group (no text messages) n=104 | Text messages with antenatal health information. No specific mHealth tools mentioned | Varied from three messages per week to daily messages | No statistically significant difference was attained between the two groups (P > 0.05). A focused group of seven participants from the intervention group was also performed and it was found out that SMS acted as a reminder and a source of motivation for the pregnant mothers | RCT with low risk of bias |
| Lund, 2012 [29] | Mobile phones as a health communication tool to improve skilled attendance at delivery in Zanzibar: a cluster-randomised controlled trial | To examine the association between a mobile phone intervention and skilled delivery attendance in a resource-limited setting | Pragmatic cluster-randomised controlled trial | Sikilled delivery | women who received SMS and mobile phone voucher componentn=1311 | standard care n=1239 | Intervention consisted of a short messaging service (SMS) and mobile phone voucher component | The frequency and content of the messages varied depending on the stage of the pregnancy. Early in the pregnancy, women received two messages a month, but, after gestational week 36, the intensity increased to two a week. | The mobile phone voucher component allowed pregnant women (mothers) to directly communicate with primary healthcare providers. This allowed access to emergency obstetric care through improved communication | RCT with low risk of bias |
| Lund, 2014 [30] | Mobile phones improve antenatal care attendance in Zanzibar: a cluster randomized controlled trial | To assess antenatal care in a comprehensive way taking into consideration utilisation of antenatal care as well as content and timing of interventions during pregnancy. | pragmatic cluster-randomised controlled trial w | Antenatal care attendance | women who received SMS and mobile phone voucher componentn=1311 | standard care n=1239 | Text message reminders and educational messages for mother delivered to mobile phone and mobile vouchers to contact health workers. Tools used: custom Wired Mothers software | Two messages per month before gestational week 36 and two messages per week after week 36 | The intervention was related with an improvement in ANC visits in the intervention group. In the intervention group, 44% of women attained four or more antenatal care visits versus 31% in the comparison group (OR, 2.39; 95% CI 1.03-5.55) | RCT with low risk of bias |
| McNabb, 2015 [31] | Assessment of the quality of antenatal care services provided by health workers using a mobile phone decision support application in northern Nigeria: a pre/post-intervention study | To assess whether the introduction of the app had an effect on the quality of antenatal care services provided by this lower-level cadre | Pre/post-intervention study | Antenatal care attendance |  |  | CommCare allows users to develop mobile phone-based decision support and data collection applications that can be used on low-end Nokia feature phones or on the Android platform. | not mentioned | Through the introduction of CommCare, the quality score improved from 13.3 at baseline to 17.2 at end line (P < 0.0001) | 5/9 |
| Mushamiri, 2015 [32] | Evaluation of the impact of a mobile health system on adherence to antenatal and postnatal care and prevention of mother-to-child transmission of HIV programs in Kenya | To demonstrate that a CHW-centered mHealth technology reminder system can improve PMTCT efforts by increasing uptake of health services. | non-randomized control group study | Antenatal care attendance and postnatal care attendance | Registered for APAS | Not registered for APAS | APAS Software | frequency vary according to the timming and visits | All CHWs communicated that APAS help them track vital events efficiently, as compared to paper based tracking system | 8/9 |
| Ngabo, 2013 [33] | Designing and Implementing an Innovative SMS-based alert system (RapidSMS-MCH) to monitor pregnancy and reduce maternal and child deaths in Rwanda | To describe requirements for designing and implementing a mobile phone-based communication system aiming at monitoring pregnancy and reducing bottlenecks in communication. | pre and post implementation | Antenatal care attendance |  |  | Electronic registration of pregnant women through text messages by community health workers (CHWs) and reminder text messages for antenatal care sent to CHWs’ mobile phones. Tools used: customized version of RapidSMS | As needed for upcoming antenatal visits and estimated delivery date | A total of 11,502 pregnancies were monitored and 362 SMS life threatening events were registered. It was found out that CHWs being more pro-active in finding new pregnant women and following them up as a result of reminders sent to the mothers. | 7/9 |
| Fedha, 2014 [34] | Impact of Mobile Telephone on Maternal Health Service Care: A Case of Njoro Division | To assess the impact of mobile technology on maternal health care services utilization and neonatal outcome in Njoro and Nesuit health Centres in Nakuru County in Kenya. | Randomized controlled trial | Antenetal care attendance | Intervention group: 191 women | Control group: 206 women | Text message reminders and educational messages for mother delivered to mobile phone. No specific mHealth tools mentioned | Appointment reminders every two weeks. Frequency of educational messages not specified | Positive association was found among women in intervention group and the number of ANC visits (96.4 % in intervention group and 92.3% in the control group, P value: 0.002) | RCT with low risk of bias |
| Adanikin, 2014 [35] | Role of reminder by text message in enhancing postnatal clinic attendance | To test the hypothesis that SMS reminders would reduce non-attendance rate at post-natal clinics in Nigeria. | non-randomized control group study | post-natal care attendance | Clinic attendance compared for intervention group and historic control group (from previous 6 months) | Clinic attendance compared for intervention group and historic control group (from previous 6 months) | Text message reminders for mother delivered to mobile phone. No specific mHealth tools mentioned | Two messages sent for each appointment: two weeks prior and 5 days prior | It was found out that the intervention group, receiving text message appointment reminders, were 50% less likely to fail to attend ( FTA) their postnatal appointment (RR of FTA 0.50; 95% CI, 0.32–0.77; 𝑃 = 0.002) | 7/9 |
